# Evaluating revised biomass equations: are some forest types more equivalent than others?

**DOI:** 10.1186/s13021-015-0042-5

**Published:** 2016-01-12

**Authors:** Coeli M. Hoover, James E. Smith

**Affiliations:** USDA Forest Service, Northern Research Station, Durham, NH USA

**Keywords:** Biomass estimation, Allometry, Forest carbon stocks, Tests of equivalence, Individual-tree estimates by species group

## Abstract

**Background:**

In 2014, Chojnacky et al. published a revised set of biomass equations for trees of temperate US forests, expanding on an existing equation set (published in 2003 by Jenkins et al.), both of which were developed from published equations using a meta-analytical approach. Given the similarities in the approach to developing the equations, an examination of similarities or differences in carbon stock estimates generated with both sets of equations benefits investigators using the Jenkins et al. (For Sci 49:12–34, 2003) equations or the software tools into which they are incorporated. We provide a roadmap for applying the newer set to the tree species of the US, present results of equivalence testing for carbon stock estimates, and provide some general guidance on circumstances when equation choice is likely to have an effect on the carbon stock estimate.

**Results:**

Total carbon stocks in live trees, as predicted by the two sets, differed by less than one percent at a national level. Greater differences, sometimes exceeding 10–15 %, were found for individual regions or forest type groups. Differences varied in magnitude and direction; one equation set did not consistently produce a higher or lower estimate than the other.

**Conclusions:**

Biomass estimates for a few forest type groups are clearly not equivalent between the two equation sets—southern pines, northern spruce-fir, and lower productivity arid western forests—while estimates for the majority of forest type groups are generally equivalent at the scales presented. Overall, the possibility of very different results between the Chojnacky and Jenkins sets decreases with aggregate summaries of those ‘equivalent’ type groups.

## Background

Nationally consistent biomass equations can be important to forest carbon research and reporting activities. In general, the consistency is based on an assumption that allometric relationships within forest species do not vary by region. Essentially, nearly identical trees even in distant locations should have nearly identical carbon mass. In 2003, Jenkins et al. published a set of 10 equations for estimating live tree biomass, developed from existing equations using a meta-analytical approach, which were intended to be applicable over temperate forests of the United States [1]. These equations were developed to support US forest carbon inventory and reporting, and had several key elements: (1) a national scale, so that regional variations in biomass estimates due to the use of local biomass equations was eliminated, (2) the exclusion of height as a predictor variable, and (3) in addition to equations to estimate aboveground biomass, a set of component equations allowing the separate estimation of biomass in coarse roots, stem bark, stem wood, and foliage. Since their introduction, these equations have been incorporated into the Fire and Fuels Extension of the Forest Vegetation Simulator as a calculation option [2], utilized in NED-2 [3], and have provided the basis for calculating the forest carbon contribution to the US annual greenhouse gas inventories for submission years 2004–2011 (e.g., see [4]). Researchers in Canada [5, 6] and the US (e.g. [7–9]) have also employed the equations while other investigators have adopted the component ratios to estimate biomass in coarse roots or other components (e.g. [10, 11]).

In 2014, Chojnacky et al. [12] introduced a revised set of generalized biomass equations for estimating aboveground biomass. These equations were developed using the same underlying data compilations and general approaches to developing the individual tree biomass estimates as for Jenkins et al. [1], but with greater differentiation among species groups, resulting in a set of 35 generalized equations: 13 for conifers, 18 for hardwoods, and 4 for woodland species. Important distinctions are: the database used to generate the revised equations was updated to include an additional 838 equations that appeared in the literature since the publication of the 2003 work or were not included at that time, taxonomic groupings were employed to account for differences in allometry, and taxa were further subdivided in cases where wood density varied considerably within a taxon. The only component equation revised by Chojnacky et al. [12] was for roots; equations were fitted for fine and coarse roots, in contrast to Jenkins et al. [1] where fine roots were not considered separately.

Based on the similarity of the equation development approach, it is likely that applications using the Jenkins et al. [1] set would have essentially the same basis for employing the revised equations. Since the primary objective of Chojnacky et al. [12] was to present the updated equations and describe the nature of the changes, only a brief discussion of the behavior of the updated equations vs. the Jenkins et al. [1] equation set was included. The authors noted that at a national level results were similar, while differences occurred in some species groups, for example, western pines, spruce/fir types, and woodland species. Given the limited information provided in Chojnacky et al. [12] we felt that a more thorough investigation of the differences in carbon stock estimates as generated with both sets of equations was needed.

One potentially practical result from a comparison of the two approaches is to identify where one set effectively substitutes for the other, which then suggests that revising or updating estimates would change little from previous analyses. For this reason we applied equivalence tests to determine the effective difference of the Chojnacky-based estimates relative to the Jenkins values. Note that hereafter we label the respective equations and species groups as Chojnacky and Jenkins (i.e., in reference to their products not the publications, per se).

In this paper, we: (1) provide a roadmap for applying the Chojnacky equations to the tree species of the US Forest Service’s forest inventory [13], (2) present results of equivalence testing for carbon stock estimates computed using both sets of equations, and (3) provide general guidance on the circumstances when the choice of equation is likely to have an important effect on the carbon stock estimate. Note that we do not attempt any evaluation of relative accuracy or the relative merit of one approach relative to the other.

## Results and discussion

**Fig. 1:**
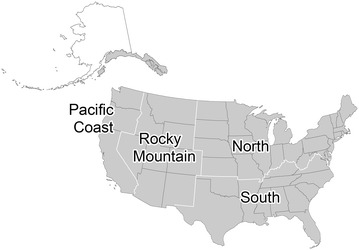
Regional classifications used in this analysis. Area of forest inventory used inclues all of conterminous United States as well as southern coastal Alaska (*shaded gray areas*)

**Fig. 2:**
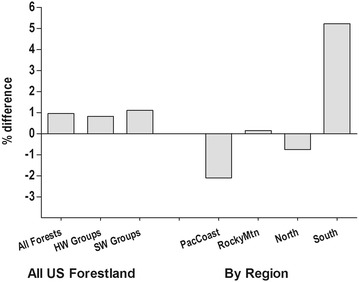
Effect of the Chojnacky et al. [12] species groups and biomass equations on estimated total stocks of carbon. Estimates are of carbon in the aboveground portion of live trees relative to the estimates provided by the Jenkins et al. [1] species groups and biomass equations

We conducted multiple equivalence tests on data aggregated at various levels of resolution. As noted by Chojnacky et al. [12], at a national level the carbon density predicted by both equations was the same when grouped by just hardwoods and softwoods, while some type groups showed differences (though no statistical comparisons were conducted). Relative differences emerged as four regions (Fig. 1) relative to the entire United States were used to summarize total carbon stocks in the aboveground portion of live trees as shown in Fig. 2. Totals for the US as well as separate summaries according to either softwood or hardwood forest type groups (not shown) are about 1 % different. This similarity in aggregate values between the two approaches holds for the Rocky Mountain and North regions, where there is less than a 1 % difference between the two. There are more sizeable differences in the Pacific Coast and South regions, notably differing in direction and magnitude. The largest difference is in the South. Note that our results are presented in terms of carbon mass rather than biomass.

To examine the drivers of those differences, we carried out equivalence tests by forest type group at both the national and regional levels on the mean density of carbon in aboveground live trees; a summary of the results is given in Table 1. The quantity tested is mean difference (Chojnacky − Jenkins) in plot level tonnes carbon per hectare; the test for equivalence was based on the percentage difference relative to the Jenkins based estimate (i.e. 100 × ((Chojnacky − Jenkins)/Jenkins)). The 5 (or 10) % of Jenkins, which was set as the equivalence interval, was put in units of tonnes per hectare for comparison with the 95 % confidence interval for the α = 0.05 (or α = 0.1) two one-sided tests (TOST) of equivalence. Of the 26 forest type groups included in the analysis, 20 are equivalent (at 5 or 10 %) at the national level, with most equivalent at 5 %. The exceptions are: spruce/fir, longleaf/slash pine, loblolly/shortleaf pine, pinyon/juniper, other western softwoods, and woodland hardwoods. At a regional level, differences emerge; in the North, only spruce/fir and loblolly/shortleaf pine are not equivalent (too few plots were available in pinyon/juniper for a reliable test statistic) while in the South, the pine types lacked equivalence, as did pinyon/juniper. This is very likely a reflection of the fact that the Chojnacky equations divide some taxa by specific gravity, while the Jenkins equations do not; softwoods generally display a larger range of specific gravity values within a species group than do hardwoods [14]. Researchers have noted considerable variability in the estimates produced by different southern pine biomass equations [15], even between different sets of local equations. Specific gravity, as mentioned above, is a factor, (southern pines exhibit considerable variability in specific gravity), as well as stand origin, and the mathematical form of the equation itself. Melson et al. [16], in their investigation of the effects of model selection on carbon stock estimates in northwest Oregon, noted that the national level Jenkins [1] equations produced biomass estimates for *Picea* that were consistently lower than from approaches developed by the investigators, and hypothesized that differences in form between *Picea* species introduced bias into the generalized equation.

**Table 1:** Mean stock of carbon in aboveground live tree biomass as computed using the equations from Jenkins et al. [1] and Chojnacky et al. [12]

Forest type group	All US^a^		North		South		Rocky Mountain		Pacific Coast	
	Jenkins	Chojnacky	Jenkins	Chojnacky	Jenkins	Chojnacky	Jenkins	Chojnacky	Jenkins	Chojnacky
White/red/jack pine	68.7**	67.2**	67.7**	66.2**	92.4**	93.5**				
Spruce/fir	45.8	40.1	47.5	41.6					20.5*	18.9*
Longleaf/slash pine	35.4	40.6			35.4	40.6				
Loblolly/shortleaf pine	47.0	54	59.0	67.1	47.2	54.1				
Pinyon/juniper	18.4	22.5	^◊^15.5	^◊^17.2	11.5	13.3	19.6	24.1	21.4	23.4
Douglas-fir	114.5*	108.0*					71.4*	66.5*	148.6*	140.9*
Ponderosa pine	50.0**	50.7**	37.3**	37.9**			46.3**	47.1**	53.5**	54.2**
Western white pine	66.2**	67.6**							^◊^74.6	^◊^76.7
Fir/spruce/mtn hemlock	92.2*	87.1*					71.8	64.4	119.4**	117.4**
Lodgepole pine	48.6**	48.2**					48.2**	47.2**	49.5**	49.7**
Hemlock/sitka spruce	155.1**	151.0**					108.8*	101.4*	159.7**	155.9**
Western larch	62.6**	65.2**					55.4**	57.5**	69.6	72.6
Redwood	236.2**	235.3**							236.2**	235.3**
Other western softwoods	27.0	35.3					*43.2* *	*45.8* *	19.5	30.4
California mixed conifer	134.7**	132.8**							134.7**	132.8**
Oak/pine	54.1**	56.6**	64.4**	65.5**	*50.9* *	*53.9* *				
Oak/hickory	72.7**	72.8**	78.7**	78.8**	65.2**	65.3**				
Oak/gum/cypress	78.1**	79.7**	86.9**	85.2**	78.5**	80.3**				
Elm/ash/cottonwood	56.6**	56.6**	60.6**	59.8**	50.4**	52.2**	48.8**	48.2**	82.3	71.8
Maple/beech/birch	80.7**	80.3**	80.1**	79.7**	82.1**	83.3**				
Aspen/birch	45.3**	43.2**	43.9**	41.8**			52.8**	50.4**	38.0**	36.5**
Alder/maple	98.5**	100.1**							99.4**	101.0**
Western oak	64.7*	61.1*							64.7**	61.1**
Tanoak/laurel	131.2**	134.6**							131.2**	134.6**
Other hardwoods	49.6**	51.2**	43.0*	45.8*	43.2*	45.9*			67.5**	66.3**
Woodland hardwoods	8.6	11.1			5.0	7.0	12.7	15.7	22.1	29.5

Values followed by a double asterisk (**) are equivalent at 5 %; values followed by a single asterisk (*) are equivalent at 10 %. Regions are as shown in Fig. 1. A diamond preceding a value indicates that the sample size was too small for a reliable test of equivalence. Data not shown for categories represented by fewer than 10 plots

^a^As shown in Fig. 1

Pinyon/juniper was not equivalent in any region in which it was tested. While fir/spruce/mountain hemlock was not equivalent in the Rocky Mountains, the stock estimates were equivalent to 5 % in the Pacific Coast region, likely a function of the species and size classes that dominate the groups in each of these regions. The elm/ash/cottonwood category is represented in each region, and was equivalent to 5 % in all areas except the Pacific Coast. The woodland class has been less well studied than the others, and so less data and fewer equations are available to construct generalized equations like those in Jenkins et al. [1] and Chojnacky et al. [12]. Consequently, the woodland equations are not equivalent at the national level or in any region.

**Fig. 3:**
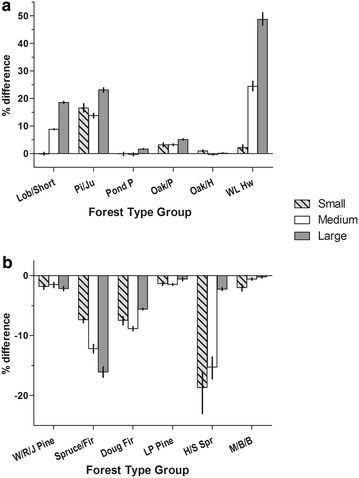
Effect of the two alternate biomass equations as relative difference in stock (*panel* **a**, positive difference,* panel* **b**, negative). Estimates are classified by forest type group and stand size class. The *error bar* represents the confidence interval used in the equivalence tests. In general, small stands have at least 50 % of stocking in small diameter trees, large stands have at least 50 % of stocking in large and medium diameter trees, with large tree stocking ≥ medium tree. The 12 forest type groups included here are: loblolly/shortleaf pine, pinyon/juniper, ponderosa pine, oak/pine, oak/hickory, and woodland hardwoods in* panel* **a**, and white/red/jack pine, spruce/fir, Douglas-fir, lodgepole pine, hemlock/Sitka spruce, and maple/beech/birch in* panel* **b**

We also explored the effect of size class on equation performance, testing each combination of forest type group and stand size class and found notable differences among size classes, though no evidence of a systematic pattern. A summary of the results is given in Fig. 3a and 3b; the error bars represent the 95 % confidence interval transformed to percentage. Not every combination is shown; groups with results similar to another or comprising a very small proportion of plots are not included. While some groups such as ponderosa pine, oak/hickory, lodgepole pine, and white/red/jack pine show small differences between size classes and are equivalent (or nearly so), others such as loblolly/shortleaf pine, longleaf/slash pine (data not shown), woodland hardwoods, and spruce/fir show a strong pattern of increasing differences with increasing stand size, with a lack of equivalence between the small and large sawtimber classes. Note that both the direction and magnitude of the differences were variable across the forest type groups. Hemlock/Sitka spruce displayed a strong trend in the opposite direction, with large differences between the two approaches for the small and medium size classes, and a very small difference in the large sawtimber class. The difference between the two sets of estimates for the woodland group that is shown in Table 1 is readily apparent in Fig. 3a, with a large increase in the percent difference as the stand size class increases. This may be due to the lack of woodland biomass equations based on diameter at root collar (drc) and the difficulty of obtaining accurate drc measurements. Bragg [17] and Bragg and McElligott [15] have discussed the importance of diameter at breast height (dbh) in some detail, comparing the performance of local, regional, and national equations for southern pines across a range of diameters. While most equations returned fairly similar estimates for trees up to 50 cm dbh, equation behavior diverged at larger diameters, in some cases returning estimates that were considerably different. In these examples, the national level Jenkins equations [1] did not produce extreme estimates, they were intermediate to those returned by local and regional equations. Melson et al. [16] also noted that considerable error could be introduced when applying equations to trees with a dbh value outside the range on which the equations were developed.

**Fig. 4:**
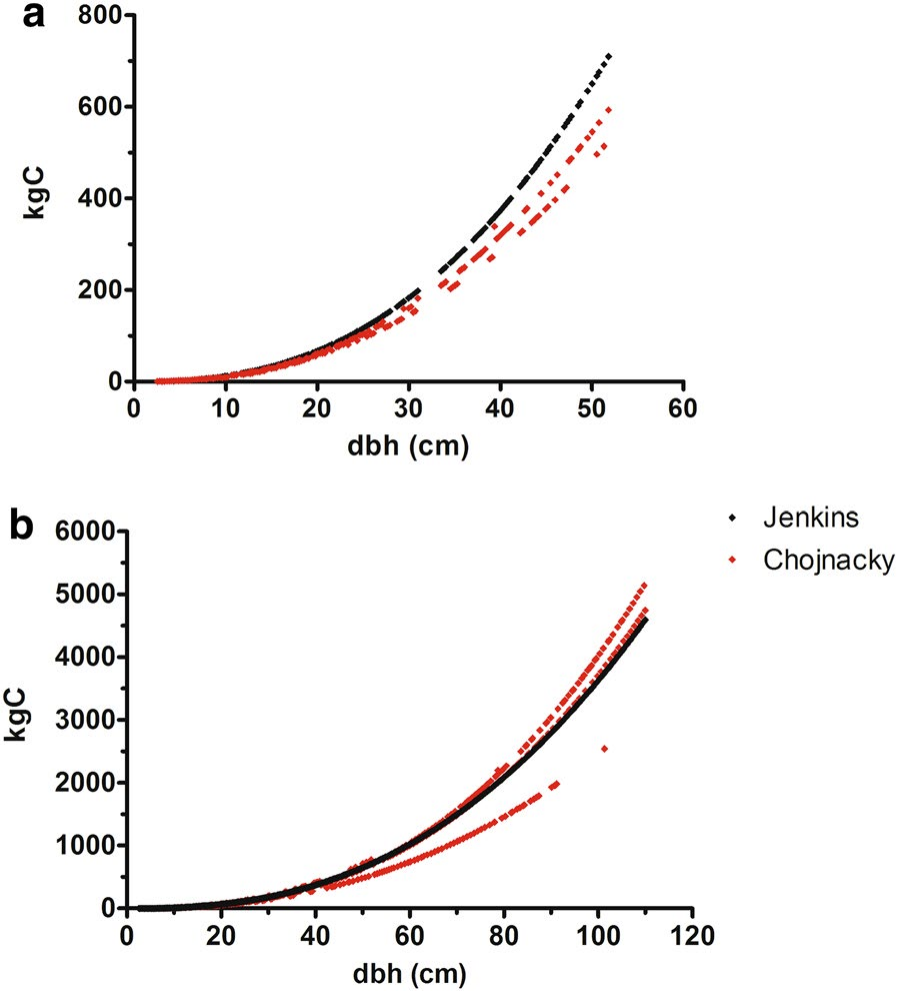
Examples of the Chojnacky-based and Jenkins-based estimates for aboveground carbon mass (kg) of individual live trees (plotted by diameter at breast height, dbh). Separate panels show the East (**a**, North and South) and the West (**b**, Pacific Coast and Rocky Mountain). This example includes trees within the fir species group of Jenkins (*black*) and their mapping to Chojnacky (*red*) species groups, which are identified in Table 2. Data points include applicable live trees in the FIADB tree data table up to the 99th percentile of diameters in the east and west, respectively

**Fig. 5:**
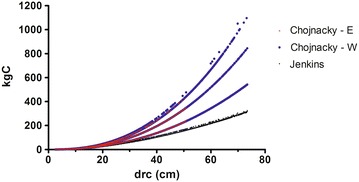
Examples of the Chojnacky-based and Jenkins-based estimates for aboveground carbon mass (kg) of individual live trees by dbh. This example includes all trees within the woodland species group of Jenkins (*black*) and their mapping to Chojnacky species groups (not identified) in the East (*red*, North and South) and the West (*blue*, Pacific Coast and Rocky Mountain). Data points include all applicable live trees in the FIADB tree data table up to the 99th percentile of diameters in the East and West, respectively

Equivalence was not tested at the level of the individual tree, though a random subset of individual tree estimates were plotted for each species group to compare tree-level biomass estimates. These plots reflect the patterns demonstrated above, with one method producing values consistently higher or lower than the other, the differences becoming more apparent at larger diameters. Tree data were also classified by east and west to further explore equation behavior within species groups where there are considerable differences in the range of tree diameters, east versus west. In many cases, no trends were revealed, but there are some key differences; a notable example is shown in Fig. 4a, b, which show the results of tree-level carbon estimates by each set of equations, categorized as east and west. In Fig. 4a, the eastern US, the Jenkins estimates are larger than those produced from the Chojnacky equations, while in Fig. 4b, the western US, the Jenkins estimates are generally somewhat lower, with the exception of the “Abies; LoSG” group. Figure 5 shows similar data for the woodland taxa; again, there is a considerable difference between the estimates computed with the two methods, with the Jenkins equations producing consistently lower estimates than the Chojnacky equations. In this case, we see no obvious differences between the predictions in the East or West.

**Fig. 6:**
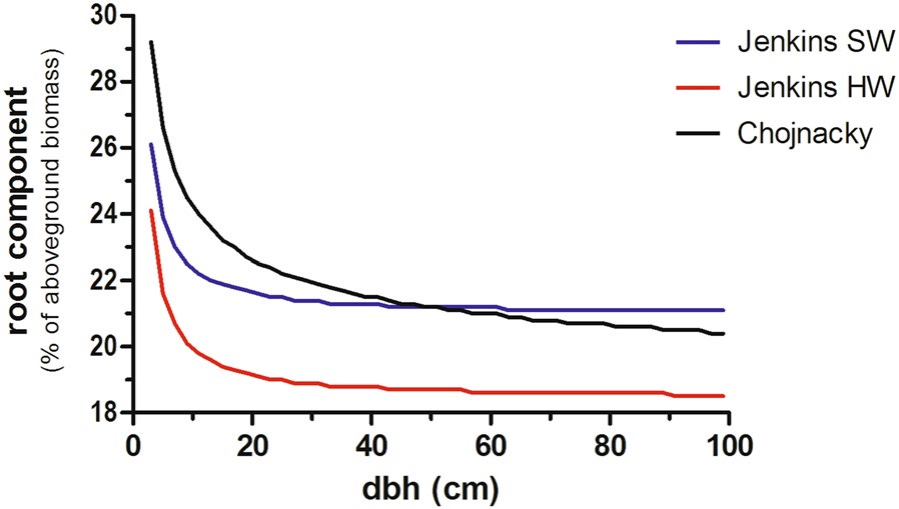
Root component by diameter of the Chojnacky-based estimates (*black*) relative to the softwood (*blue*) and hardwood (*red*) root components of the Jenkins-based estimates. Root biomass is calculated as equal to a proportion of aboveground biomass

As mentioned above, the belowground component equations were also revised in the 2014 publication, and while not divided according to hardwood and softwood, the revised root component equations are subdivided by coarse and fine roots. There are important differences in the shape of the root component curve between the two approaches (Fig. 6), and the Jenkins hardwood equation yields a consistently lower proportion than the Chojnacky equation. This suggests that adopting the Chojnacky estimates for full above- and belowground tree would add up to an additional 2–3 % of biomass for hardwoods but would also affect some softwood estimates. A preliminary analysis did show an effect on the test for the 5 % equivalence for some categories. However, our emphases here are the various species groups/equations and not the components.

## Conclusions

The revised approach to developing these biomass equations has the effect of providing better regional differentiation/representation at the plot/stand level summaries by allowing for separation within the taxonomic classes according to wood properties or growth habit. The emergence of Southern pines as distinctly different under the Chojnacky groups is one example. It is challenging to provide specific criteria for choosing one set of equations over the other, since validating any biomass equation requires the destructive sampling of multiple stems across a range of diameters. The Chojnacky groups appear to provide greater resolution across forest types and regions. From this, investigators working in southern pine, northern spruce-fir, pinyon-juniper, and woodland types may be advised to use the updated equations [12], which provide more taxonomic resolution. It should also be noted that estimates of change over time are somewhat less sensitive to equation choice than stock estimates, so if change is the primary variable of interest, the user can select either equation set, based on personal preference.

Individual large diameter trees can be very different—Chojnacky relative to Jenkins—given the general trends of the tree-level estimates (Figs. 4 and 5 in this manuscript as well as Figs. 2, 3, and 4 in Chojnacky et al. [12]). This effect of one or a very few larger trees can result in very different estimates even in an “equivalent” forest type group, and this potential for larger differences is reflected in plot-level data. For example, in some eastern hardwood type groups, which were consistently identified as equivalent, up to one-third of the plots were individually more than 5 % different. The oak/gum/cypress type group in the South had 8 % of the plots with greater carbon density by over 5 % with the Jenkins estimates, while 27 % of plots had over 5 % greater carbon. The remaining 65 % of the individual plots are within the 5 % bounds (data not shown here). This is consistent with our observation about similarities between the two sets and scale (Fig. 2)—the sometimes obvious and large differences for some forest type groups (all scales) become obscured when summed to total live tree carbon for the US. Singling out the correct or most accurate equations is beyond the scope here; however, caution is always warranted when applying equations to trees that are considerably outside the range of diameters used to construct the equations [16].

Our results point to a few forest type groups that are clearly not equivalent—southern pines, northern spruce-fir, and lower productivity arid western forests—while the majority of forest type groups are generally equivalent at the scales presented. Overall, the possibility of very different results between the Chojnacky and Jenkins sets decreases with aggregate summaries of those ‘equivalent’ type groups.

## Methods

### Tree data source

In order to implement the revised biomass equations and identify applications where they are effectively interchangeable, or equivalent, we used the Forest Inventory and Analysis Data Base (FIADB) compiled by the Forest Inventory and Analysis (FIA) Program of the US Forest Service [13]. The data are based on continuous systematic annualized sampling of US forest lands, which are then compiled and made available by the FIA program of the US Forest Service [18]; the specific data in use here were downloaded from http://apps.fs.fed.us/fiadb-downloads/datamart.html on 02 June 2015. Surveys are organized and conducted on a large system of permanent plots over all land within individual states so that a portion of the survey data is collected each year on a continuous cycle, with remeasurement at 5 or 10 years depending on the state. The portion of the data used here include the conterminous United States (i.e., 48 states), and the portion of southern coastal Alaska that has the established permanent annual survey plots (the gray areas in Fig. 1).

Our focus here is on the tree data of the FIADB, and for this analysis we present the Chojnacky and Jenkins estimates in terms of carbon mass (i.e., kg carbon per tree or tonnes per hectare per plot). We use the entire tree data table to assure that all applicable species (the gray areas in Fig. 1) are represented. All other summaries are based on the most recent (most up-to-date) set of tree and plot data available per state, with the Chojnacky and Jenkins estimates expressed as tonnes of carbon per hectare in live trees on forest inventory plots. These plot-level values are expanded to population totals, that is, total carbon stock per state, as provided within the FIADB as the basis for the result presented in Fig. 2. A subset of the current forest plot level summaries where the entire plot is identified as forested (i.e., single condition forest plots) is the basis for the results provided in Table 1 and Fig. 3.

### Application of Chojnacky et al. [12] to the FIADB

Chojnacky et al. [12] provided a revised and expanded set of biomass equations following the approach of Jenkins et al. [1]. The revised equations are based on an approach similar to that of Jenkins et al. [1] and with an expanded database of published biomass equations; see Chojnacky et al. [12] for details. The new set of 35 Chojnacky species groups are based on taxon (family or genera), growth habit, or average wood density. See Table 2 for the links between species in the FIADB and the Jenkins and Chojnacky classifications. This allocation to the newer categories is not a simple mapping of the 10 Jenkins groups to Chojnacky groups. That is, while Jenkins groups are split among Chojnacky groups, so also the Chojnacky groups are in some cases composed of species from different Jenkins groups. While Chojnacky et al. [12] developed the set of new groups based on the FIADB, similar to Jenkins et al. [1], a very small percentage of hardwood species were not explicitly named (i.e., families were not listed [12]). We assigned these to the “Cor/Eri/Lau/Etc” group (Table 2).

**Table 2:** Guide to applying Chojnacky species groups (as shown in Table 5, Chojnacky et al. [12]) to US species

Scientific name	Common name	Jenkins group	Chojnacky et al. parameters when diameter is measured at	
			Breast height	Root collar
*Abies spp.*	Fir spp.	T Fir/Hem	Abies; HiSG	Pinac; WL
*A. amabilis*	Pacific silver fir	T Fir/Hem	Abies; HiSG	Pinac; WL
*A. balsamea*	Balsam fir	T Fir/Hem	Abies; LoSG	Pinac; WL
*A. bracteata*	Bristlecone fir	T Fir/Hem	Abies; HiSG	Pinac; WL
*A. concolor*	White fir	T Fir/Hem	Abies; HiSG	Pinac; WL
*A. fraseri*	Fraser fir	T Fir/Hem	Abies; HiSG	Pinac; WL
*A. grandis*	Grand fir	T Fir/Hem	Abies; HiSG	Pinac; WL
*A. lasiocarpa var. arizonica*	Corkbark fir	T Fir/Hem	Abies; HiSG	Pinac; WL
*A. lasiocarpa*	Subalpine fir	T Fir/Hem	Abies; LoSG	Pinac; WL
*A. magnifica*	California red fir	T Fir/Hem	Abies; HiSG	Pinac; WL
*A. shastensis*	Shasta red fir	T Fir/Hem	Abies; HiSG	Pinac; WL
*A. procera*	Noble fir	T Fir/Hem	Abies; HiSG	Pinac; WL
*Chamaecyparis spp.*	White-cedar spp.	Cedar/Larch	Cupr; MedSG	Cupre; WL
*C. lawsoniana*	Port Orford cedar	Cedar/Larch	Cupr; MedSG	Cupre; WL
*C. nootkatensi*	Alaska yellow cedar	Cedar/Larch	Cupr; HiSG	Cupre; WL
*C. thyoides*	Atlantic white cedar	Cedar/Larch	Cupr; MedSG	Cupre; WL
*Cupressus spp.*	Cypress	Woodland	Cupr; HiSG	Cupre; WL
*C. arizonica*	Arizona cypress	Woodland	Cupr; HiSG	Cupre; WL
*C. bakeri*	Baker/Modoc cypress	Woodland	Cupr; HiSG	Cupre; WL
*C. forbesii*	Tecate cypress	Woodland	Cupr; HiSG	Cupre; WL
*C. macrocarpa*	Monterey cypress	Woodland	Cupr; HiSG	Cupre; WL
*C. sargentii*	Sargent’s cypress	Woodland	Cupr; HiSG	Cupre; WL
*C. macnabiana*	MacNab’s cypress	Woodland	Cupr; HiSG	Cupre; WL
*Juniperus spp.*	Redcedar/juniper spp.	Cedar/Larch	Cupr; HiSG	Cupre; WL
*J. pinchotii*	Pinchot juniper	Woodland	Cupr; HiSG	Cupre; WL
*J. coahuilensis*	Redberry juniper	Woodland	Cupr; HiSG	Cupre; WL
*J. flaccida*	Drooping juniper	Woodland	Cupr; HiSG	Cupre; WL
*J. ashei*	Ashe juniper	Woodland	Cupr; HiSG	Cupre; WL
*J. californica*	California juniper	Woodland	Cupr; HiSG	Cupre; WL
*J. deppeana*	Alligator juniper	Woodland	Cupr; HiSG	Cupre; WL
*J. occidentalis*	Western juniper	Woodland	Cupr; HiSG	Cupre; WL
*J. osteosperma*	Utah juniper	Woodland	Cupr; HiSG	Cupre; WL
*J. scopulorum*	Rocky Mtn. juniper	Woodland	Cupr; HiSG	Cupre; WL
*J. virginiana var. silcicola*	Southern redcedar	Cedar/Larch	Cupr; HiSG	Cupre; WL
*J. virginiana*	Easterm redcedar	Cedar/Larch	Cupr; HiSG	Cupre; WL
*J. monosperma*	Oneseed juniper	Woodland	Cupr; HiSG	Cupre; WL
*Larix spp.*	Larch spp.	Cedar/Larch	Larix	Pinac; WL
*L. laricina*	Tamarack	Cedar/Larch	Larix	Pinac; WL
*L. lyallii*	Subalpine larch	Cedar/Larch	Larix	Pinac; WL
*L. occidentalis*	Western larch	Cedar/Larch	Larix	Pinac; WL
*Calocedrus decurrens*	Incense-cedar	Cedar/Larch	Cupr; MedSG	Cupre; WL
*Picea spp.*	Spruce spp.	Spruce	Pice; HiSG	Pinac; WL
*P. abies*	Norway spruce	Spruce	Pice; HiSG	Pinac; WL
*P. breweriana*	Brewer spruce	Spruce	Pice; HiSG	Pinac; WL
*Picea engelmannii*	Englemann spruce	Spruce	Pice; LoSG	Pinac; WL
*P. glauca*	White spruce	Spruce	Pice; HiSG	Pinac; WL
*P. mariana*	Black spruce	Spruce	Pice; HiSG	Pinac; WL
*P. pungens*	Blue spruce	Spruce	Pice; HiSG	Pinac; WL
*P. rubens*	Red spruce	Spruce	Pice; HiSG	Pinac; WL
*P. sitchensis*	Sitka spruce	Spruce	Pice; LoSG	Pinac; WL
*Pinus spp.*	Pine spp.	Pine	Pinu; LoSG	Pinac; WL
*P. albicaulis*	Whitebark pine	Pine	Pinu; LoSG	Pinac; WL
*P. aristata*	Rocky Mtn. bristlecone pine	Pine	Pinu; LoSG	Pinac; WL
*P. attenuata*	Knobcone pine	Pine	Pinu; LoSG	Pinac; WL
*P. balfouriana*	Foxtail pine	Pine	Pinu; LoSG	Pinac; WL
*P. banksiana*	Jack pine	Pine	Pinu; LoSG	Pinac; WL
*P. edulis*	Common/two-needle pinyon	Pine	Pinu; HiSG	Pinac; WL
*P. clausa*	Sand pine	Pine	Pinu; HiSG	Pinac; WL
*P. contorta*	Lodgepole pine	Pine	Pinu; LoSG	Pinac; WL
*P. coulteri*	Coulter pine	Pine	Pinu; LoSG	Pinac; WL
*P. echinata*	Shortleaf pine	Pine	Pinu; HiSG	Pinac; WL
*P. elliottii*	Slash pine	Pine	Pinu; HiSG	Pinac; WL
*P. engelmannii*	Apache pine	Pine	Pinu; LoSG	Pinac; WL
*P. flexilis*	Limber pine	Pine	Pinu; LoSG	Pinac; WL
*P. strobiformis*	Southwestern white pine	Pine	Pinu; LoSG	Pinac; WL
*P. glabra*	Spruce pine	Pine	Pinu; LoSG	Pinac; WL
*P. jeffreyi*	Jeffrey pine	Pine	Pinu; LoSG	Pinac; WL
*P. lambertiana*	Sugar pine	Pine	Pinu; LoSG	Pinac; WL
*P. leiophylla*	Chihauhua pine	Pine	Pinu; LoSG	Pinac; WL
*P. monticola*	Western white pine	Pine	Pinu; LoSG	Pinac; WL
*P. muricata*	Bishop pine	Pine	Pinu; HiSG	Pinac; WL
*P. palustris*	Longleaf pine	Pine	Pinu; HiSG	Pinac; WL
*P. ponderosa*	Ponderosa pine	Pine	Pinu; LoSG	Pinac; WL
*P. pungens*	Table Mountain pine	Pine	Pinu; HiSG	Pinac; WL
*P. radiata*	Monterey pine	Pine	Pinu; LoSG	Pinac; WL
*P. resinosa*	Red pine	Pine	Pinu; LoSG	Pinac; WL
*P. rigida*	Pitch pine	Pine	Pinu; HiSG	Pinac; WL
*P. sabiniana*	Gray pine	Pine	Pinu; LoSG	Pinac; WL
*P. serotina*	Pond pine	Pine	Pinu; HiSG	Pinac; WL
*P. strobus*	Eastern white pine	Pine	Pinu; LoSG	Pinac; WL
*P. sylvestris*	Scotch pine	Pine	Pinu; LoSG	Pinac; WL
*P. taeda*	Loblolly pine	Pine	Pinu; HiSG	Pinac; WL
*P. virginiana*	Viginia pine	Pine	Pinu; HiSG	Pinac; WL
*P. monophylla*	Singleleaf pinyon	Pine	Pinu; LoSG	Pinac; WL
*P. discolor*	Border pinyon	Pine	Pinu; LoSG	Pinac; WL
*P. arizonica*	Arizona pine	Pine	Pinu; LoSG	Pinac; WL
*P. nigra*	Austrian pine	Pine	Pinu; LoSG	Pinac; WL
*P. washoensis*	Washoe pine	Pine	Pinu; LoSG	Pinac; WL
*P. quadrifolia*	Four leaf pine	Pine	Pinu; LoSG	Pinac; WL
*P. torreyana*	Torrey pine	Pine	Pinu; LoSG	Pinac; WL
*P. cembroides*	Mexican pinyon pine	Pine	Pinu; LoSG	Pinac; WL
*P. remota*	Papershell pinyon pine	Pine	Pinu; LoSG	Pinac; WL
*P. longaeva*	Great Basin bristlecone pine	Pine	Pinu; LoSG	Pinac; WL
*P. monophylla var. fallax*	Arizona pinyon pine	Pine	Pinu; LoSG	Pinac; WL
*P. elliottii var. elliottii*	Honduras pine	Pine	Pinu; LoSG	Pinac; WL
*Pseudotsuga spp.*	Douglas-fir spp.	Doug Fir	Pseud	Pinac; WL
*P. macrocarpa*	Bigcone Douglas-fir	Doug Fir	Pseud	Pinac; WL
*P. menziesii*	Douglas-fir	Doug Fir	Pseud	Pinac; WL
*Sequoia sempervirens*	Redwood	Cedar/Larch	Cupr; MedSG	Cupre; WL
*Sequoiadendron giganteum*	Giant sequoia	Cedar/Larch	Cupr; MedSG	Cupre; WL
*Taxodium spp.*	Baldcypress spp.	Cedar/Larch	Cupr; HiSG	Cupre; WL
*T. distichum*	Baldcypress	Cedar/Larch	Cupr; HiSG	Cupre; WL
*T. ascendens*	Pondcypress	Cedar/Larch	Cupr; HiSG	Cupre; WL
*T. mucronatum*	Montezuma baldcypress	Cedar/Larch	Cupr; HiSG	Cupre; WL
*Taxus spp.*	Yew spp.	T Fir/Hem	Pseud	
*T. brevifolia*	Pacific yew	T Fir/Hem	Pseud	
*T. floridana*	Florida yew	T Fir/Hem	Pseud	
*Thuja spp.*	Thuja spp.	Cedar/Larch	Cupr; MedSG	Cupre; WL
*T. occidentalis*	Northern white-cedar	Cedar/Larch	Cupr; LoSG	Cupre; WL
*T. plicata*	Western redcedar	Cedar/Larch	Cupr; MedSG	Cupre; WL
*Torreya spp.*	Torreya (nutmeg) spp.	T Fir/Hem	Pseud	
*T. californica*	California torreya	T Fir/Hem	Pseud	
*T. taxifolia*	Florida torreya	T Fir/Hem	Pseud	
*Tsuga spp.*	Hemlock spp.	T Fir/Hem	Tsug; HiSG	Pinac; WL
*T. canadensis*	Eastern hemlock	T Fir/Hem	Tsug; LoSG	Pinac; WL
*T. caroliniana*	Carolina hemlock	T Fir/Hem	Tsug; HiSG	Pinac; WL
*T. heterophylla*	Western hemlock	T Fir/Hem	Tsug; HiSG	Pinac; WL
*T. mertensiana*	Mountain hemlock	T Fir/Hem	Tsug; HiSG	Pinac; WL
*Dead conifer*	Unknown dead conifer	Pine	Pinu; LoSG	
*Acacia spp.*	Acacia spp.	Woodland	Fab/Jug	Fab/Ros; WL
*A. farnesiana*	Sweet acacia	Woodland	Fab/Jug	Fab/Ros; WL
*A. greggii*	Catclaw acacia	Woodland	Fab/Jug	Fab/Ros; WL
*Acer spp.*	Maple spp.	S Maple/Bir	Acer; LoSG	
*A. barbatum*	Florida maple	S Maple/Bir	Acer; HiSG	
*A. macrophyllum*	Bigleaf maple	S Maple/Bir	Acer; LoSG	
*A. negundo*	Boxelder	S Maple/Bir	Acer; LoSG	
*A. nigrum*	Black maple	H Maple/Oak	Acer; HiSG	
*A. pensylvanicum*	Striped maple	S Maple/Bir	Acer; LoSG	
*A. rubrum*	Red maple	S Maple/Bir	Acer; LoSG	
*A. saccharinum*	Silver maple	S Maple/Bir	Acer; LoSG	
*A. saccharum*	Sugar maple	H Maple/Oak	Acer; HiSG	
*A. spicatum*	Mountain maple	S Maple/Bir	Acer; LoSG	
*A. platanoides*	Norway maple	S Maple/Bir	Acer; LoSG	
*A. glabrum*	Rocky Mtn. maple	Woodland	Acer; LoSG	
*A. grandidentatum*	Bigtooth maple	Woodland	Acer; LoSG	
*A. leucoderme*	Chalk maple	Mixed HW	Acer; LoSG	
*Aesculus spp.*	Buckeye spp.	Mixed HW	Hip/Til	
*A.glabra*	Ohio buckeye	Mixed HW	Hip/Til	
*A.flava*	Yellow buckeye	Mixed HW	Hip/Til	
*A.californica*	California buckeye	Mixed HW	Hip/Til	
*A.glabra var. arguta*	Texas buckeye	Mixed HW	Hip/Til	
*A.pavia*	Red buckeye	Mixed HW	Hip/Til	
*A.sylvatica*	Painted buckeye	Mixed HW	Hip/Til	
*Ailanthus altissima*	Ailanthus	Mixed HW	Cor/Eri/Lau/Etc	
*Albizia julibrissin*	Mimosa/silktree	Mixed HW	Fab/Jug	Fab/Ros; WL
*Alnus spp.*	Alder spp.	Aspen/Alder	Betu; LoSG	
*A. rubra*	Red alder	Aspen/Alder	Betu; LoSG	
*A. rhombifolia*	White alder	Aspen/Alder	Betu; LoSG	
*A. oblongifolia*	Arizona alder	Aspen/Alder	Betu; LoSG	
*A. glutinosa*	European alder	Aspen/Alder	Betu; LoSG	
*Amelanchier spp.*	Serviceberry spp.	Mixed HW	Cor/Eri/Lau/Etc	Fab/Ros; WL
*A. arborea*	Common serviceberry	Mixed HW	Cor/Eri/Lau/Etc	Fab/Ros; WL
*A. sanguinea*	Roundleaf serviceberry	Mixed HW	Cor/Eri/Lau/Etc	Fab/Ros; WL
*Arbutus spp.*	Madrone spp.	Mixed HW	Cor/Eri/Lau/Etc	
*A. menziesii*	Pacific madrone	Mixed HW	Cor/Eri/Lau/Etc	
*A. arizonica*	Arizona madrone	Mixed HW	Cor/Eri/Lau/Etc	
*A. xalapensis*	Texas madrone	Mixed HW	Cor/Eri/Lau/Etc	
*Asimina triloba*	Pawpaw	Mixed HW	Cor/Eri/Lau/Etc	
*Betula spp.*	Birch spp.	S Maple/Bir	Betu; Med1SG	
*B. alleghaniensis*	Yellow birch	S Maple/Bir	Betu; Med2SG	
*B. lenta*	Sweet birch	S Maple/Bir	Betu; HiSG	
*B. nigra*	River birch	S Maple/Bir	Betu; Med1SG	
*B. occidentalis*	Water birch	S Maple/Bir	Betu; Med2SG	
*B. papyrifera*	Paper birch	S Maple/Bir	Betu; Med1SG	
*B. uber*	Virginia roundleaf birch	S Maple/Bir	Betu; Med2SG	
*B. utahensis*	Northwestern paper birch	S Maple/Bir	Betu; Med2SG	
*B. populifolia*	Gray birch	S Maple/Bir	Betu; Med1SG	
*Sideroxylon lanuginosum*	Chittamwood/gum bumelia	Mixed HW	Cor/Eri/Lau/Etc	
*Carpinus caroliniana*	American hornbeam	Mixed HW	Betu; Med2SG	
*Carya spp.*	Hickory spp.	H Maple/Oak	Fab/Jug/Carya	
*C. aquatica*	Water hickory	H Maple/Oak	Fab/Jug/Carya	
*C. cordiformis*	Bitternut hickory	H Maple/Oak	Fab/Jug/Carya	
*C. glabra*	Pignut hickory	H Maple/Oak	Fab/Jug/Carya	
*C. illinoinensis*	Pecan	H Maple/Oak	Fab/Jug/Carya	
*C. laciniosa*	Shellbark hickory	H Maple/Oak	Fab/Jug/Carya	
*C. myristiciformis*	Nutmeg hickory	H Maple/Oak	Fab/Jug/Carya	
*C. ovata*	Shagbark hickory	H Maple/Oak	Fab/Jug/Carya	
*C. texana*	Black hickory	H Maple/Oak	Fab/Jug/Carya	
*C. alba*	Mockernut hickory	H Maple/Oak	Fab/Jug/Carya	
*C. pallida*	Sand hickory	H Maple/Oak	Fab/Jug/Carya	
*C. floridana*	Scrub hickory	H Maple/Oak	Fab/Jug/Carya	
*C. ovalis*	Red hickory	H Maple/Oak	Fab/Jug/Carya	
*C. carolinae*-*septentrionalis*	Southern shagbark hickory	H Maple/Oak	Fab/Jug/Carya	
*Castanea spp.*	Chestnut spp.	Mixed HW	Faga; Decid	Fagac; WL
*C. dentata*	American chestnut	Mixed HW	Faga; Decid	Fagac; WL
*C. pumila*	Allegheny chinkapin	Mixed HW	Faga; Decid	Fagac; WL
*C. pumila var. ozarkensis*	Ozark chinkapin	Mixed HW	Faga; Decid	Fagac; WL
*C. mollissima*	Chinese chestnut	Mixed HW	Faga; Decid	Fagac; WL
*Chrysolepis chrysophylla*	Giant/golden chinkapin	Mixed HW	Faga; Evergrn	Fagac; WL
*Catalpa spp.*	Catalpa spp.	Mixed HW	Cor/Eri/Lau/Etc	
*C. bignonioide*	Southern catalpa	Mixed HW	Cor/Eri/Lau/Etc	
*C. speciosa*	Northern catalpa	Mixed HW	Cor/Eri/Lau/Etc	
*Celtis*	Hackberry spp.	Mixed HW	Cor/Eri/Lau/Etc	
*C. laevigata*	Sugarberry	Mixed HW	Cor/Eri/Lau/Etc	
*C. occidentalis*	Hackberry	Mixed HW	Cor/Eri/Lau/Etc	
*C. laevigata var. reticulata*	Netleaf hackberry	Mixed HW	Cor/Eri/Lau/Etc	
*Cercis canadensis*	Eastern redbud	Mixed HW	Fab/Jug	Fab/Ros; WL
*Cercocarpus ledifoliu*	Curlleaf mountain-mahogany	Woodland	Cor/Eri/Lau/Etc	Fab/Ros; WL
*Cladrastis kentukea*	Yellowwood	Mixed HW	Fab/Jug	Fab/Ros; WL
*Cornus spp.*	Dogwood spp.	Mixed HW	Cor/Eri/Lau/Etc	
*C. florida*	Flowering dogwood	Mixed HW	Cor/Eri/Lau/Etc	
*C. nuttallii*	Pacific dogwood	Mixed HW	Cor/Eri/Lau/Etc	
*Crataegus spp.*	Hawthorn spp.	Mixed HW	Cor/Eri/Lau/Etc	Fab/Ros; WL
*C. crusgalli*	Cockspur hawthorn	Mixed HW	Cor/Eri/Lau/Etc	Fab/Ros; WL
*C. mollis*	Downy hawthorn	Mixed HW	Cor/Eri/Lau/Etc	Fab/Ros; WL
*C. brainerdii*	Brainerd’s hawthorn	Mixed HW	Cor/Eri/Lau/Etc	Fab/Ros; WL
*C. calpodendron*	Pear hawthorn	Mixed HW	Cor/Eri/Lau/Etc	Fab/Ros; WL
*C. chrysocarpa*	Fireberry hawthorn	Mixed HW	Cor/Eri/Lau/Etc	Fab/Ros; WL
*C. dilatata*	Broadleaf hawthorn	Mixed HW	Cor/Eri/Lau/Etc	Fab/Ros; WL
*C. flabellata*	Fanleaf hawthorn	Mixed HW	Cor/Eri/Lau/Etc	Fab/Ros; WL
*C. monogyna*	Oneseed hawthorn	Mixed HW	Cor/Eri/Lau/Etc	Fab/Ros; WL
*C. pedicellata*	Scarlet hawthorn	Mixed HW	Cor/Eri/Lau/Etc	Fab/Ros; WL
*Eucalyptus spp.*	Eucalyptus spp.	Mixed HW	Cor/Eri/Lau/Etc	
*E. globulus*	Tasmanian bluegum	Mixed HW	Cor/Eri/Lau/Etc	
*E. camaldulensi*	River redgum	Mixed HW	Cor/Eri/Lau/Etc	
*E. grandis*	Grand eucalyptus	Mixed HW	Cor/Eri/Lau/Etc	
*E. robusta*	Swamp mahogany	Mixed HW	Cor/Eri/Lau/Etc	
*Diospyros spp.*	Persimmon spp.	Mixed HW	Cor/Eri/Lau/Etc	
*D. virginiana*	Common persimmon	Mixed HW	Cor/Eri/Lau/Etc	
*D. texana*	Texas persimmon	Mixed HW	Cor/Eri/Lau/Etc	
*Ehretia anacua*	Anacua knockaway	Mixed HW	Cor/Eri/Lau/Etc	
*Fagus grandifolia*	American beech	H Maple/Oak	Faga; Decid	Fagac; WL
*Fraxinus spp.*	Ash spp.	Mixed HW	Olea; LoSG	
*F. americana*	White ash	Mixed HW	Olea; HiSG	
*F. latifolia*	Oregon ash	Mixed HW	Olea; LoSG	
*F. nigra*	Black ash	Mixed HW	Olea; LoSG	
*F. pennsylvanica*	Green ash	Mixed HW	Olea; LoSG	
*F. profunda*	Pumpkin ash	Mixed HW	Olea; LoSG	
*F. quadrangulata*	Blue ash	Mixed HW	Olea; LoSG	
*F. velutina*	Velvet ash	Mixed HW	Olea; LoSG	
*F. caroliniana*	Carolina ash	Mixed HW	Olea; LoSG	
*F. texensis*	Texas ash	Mixed HW	Olea; LoSG	
*Gleditsia spp.*	Honeylocust spp.	Mixed HW	Fab/Jug	Fab/Ros; WL
*G. aquatica*	Waterlocust	Mixed HW	Fab/Jug	Fab/Ros; WL
*G. triacanthos*	Honeylocust	Mixed HW	Fab/Jug	Fab/Ros; WL
*Gordonia lasianthus*	Loblolly-bay	Mixed HW	Cor/Eri/Lau/Etc	
*Ginkgo biloba*	Ginkgo	Mixed HW	Cor/Eri/Lau/Etc	
*Gymnocluadus diocicus*	Kentucky coffeetree	Mixed HW	Fab/Jug	Fab/Ros; WL
*Halesia spp.*	Silverbell spp.	Mixed HW	Cor/Eri/Lau/Etc	
*H. carolina*	Carolina silverbell	Mixed HW	Cor/Eri/Lau/Etc	
*H. diptera*	Two-wing silverbell	Mixed HW	Cor/Eri/Lau/Etc	
*H. parviflora*	Little silverbell	Mixed HW	Cor/Eri/Lau/Etc	
*Ilex opaca*	American holly	Mixed HW	Cor/Eri/Lau/Etc	
*Juglans spp.*	Walnut spp.	Mixed HW	Fab/Jug	
*J. cinerea*	Butternut	Mixed HW	Fab/Jug	
*J. nigra*	Black walnut	Mixed HW	Fab/Jug	
*J. hindsii*	No. California black walnut	Mixed HW	Fab/Jug	
*J. californica*	So. California black walnut	Mixed HW	Fab/Jug	
*J. microcarpa*	Texas walnut	Mixed HW	Fab/Jug	
*J. major*	Arizona walnut	Mixed HW	Fab/Jug	
*Liquidambar styraciflua*	Sweetgum	Mixed HW	Hama	
*Liriodendron tulipifera*	Yellow poplar	Mixed HW	Magno	
*Lithocarpus densiflorus*	Tanoak	Mixed HW	Faga; Evergrn	Fagac; WL
*Maclura pomifera*	Osage orange	Mixed HW	Cor/Eri/Lau/Etc	
*Magnolia spp.*	Magnolia spp.	Mixed HW	Magno	
*M. acuminata*	Cucumbertree	Mixed HW	Magno	
*M. grandiflora*	Southern magnolia	Mixed HW	Magno	
*M. virginiana*	Sweeetbay	Mixed HW	Magno	
*M. macrophylla*	Bigleaf magnolia	Mixed HW	Magno	
*M. fraseri*	Mountain/Frasier magnolia	Mixed HW	Magno	
*M. pyramidata*	Pyramid magnolia	Mixed HW	Magno	
*M. tripetala*	Umbrella magnolia	Mixed HW	Magno	
*Malus spp.*	Apple spp.	Mixed HW	Cor/Eri/Lau/Etc	Fab/Ros; WL
*M. fusca*	Oregon crab apple	Mixed HW	Cor/Eri/Lau/Etc	Fab/Ros; WL
*M. angustifolia*	Southern crabapple	Mixed HW	Cor/Eri/Lau/Etc	Fab/Ros; WL
*M. coronaria*	Sweet crabapple	Mixed HW	Cor/Eri/Lau/Etc	Fab/Ros; WL
*M. ioensi*	Prairie crabapple	Mixed HW	Cor/Eri/Lau/Etc	Fab/Ros; WL
*Morus spp.*	Mulberry spp.	Mixed HW	Cor/Eri/Lau/Etc	
*M. alba*	White mulberry	Mixed HW	Cor/Eri/Lau/Etc	
*M. rubra*	Red mulberry	Mixed HW	Cor/Eri/Lau/Etc	
*M. microphyll*	Texas mulberry	Mixed HW	Cor/Eri/Lau/Etc	
*M. nigra*	Black mulberry	Mixed HW	Cor/Eri/Lau/Etc	
*Nyssa spp.*	Tupelo spp.	Mixed HW	Cor/Eri/Lau/Etc	
*N. aquatica*	Water tupelo	Mixed HW	Cor/Eri/Lau/Etc	
*N. ogeche*	Ogeechee tupelo	Mixed HW	Cor/Eri/Lau/Etc	
*N. sylvatica*	Blackgum	Mixed HW	Cor/Eri/Lau/Etc	
*N. biflora*	Swamp tupelo	Mixed HW	Cor/Eri/Lau/Etc	
*Ostrya virginiana*	Eastern hophornbeam	Mixed HW	Betu; HiSG	
*Oxydendrum arboreum*	Sourwood	Mixed HW	Cor/Eri/Lau/Etc	
*Paulownia tomentosa*	Paulownia/empress tree	Mixed HW	Cor/Eri/Lau/Etc	
*Persea spp.*	Bay spp.	Mixed HW	Cor/Eri/Lau/Etc	
*Persea borbonia*	Redbay	Mixed HW	Cor/Eri/Lau/Etc	
*Planera aquatica*	Water elm/planetree	Mixed HW	Cor/Eri/Lau/Etc	
*Platanus spp.*	Sycamore spp.	Mixed HW	Cor/Eri/Lau/Etc	
*P. racemosa*	California sycamore	Mixed HW	Cor/Eri/Lau/Etc	
*P. occidentalis*	American sycamore	Mixed HW	Cor/Eri/Lau/Etc	
*P. wrightii*	Arizona sycamore	Mixed HW	Cor/Eri/Lau/Etc	
*Populus spp.*	Cottonwood/poplar spp.	Aspen/Alder	Sali; HiSG	
*P. balsamifera*	Balsam poplar	Aspen/Alder	Sali; LoSG	
*P. deltoides*	Eastern cottonwood	Aspen/Alder	Sali; HiSG	
*P. grandidentata*	Bigtooth aspen	Aspen/Alder	Sali; HiSG	
*P. heterophylla*	Swamp cottonwood	Aspen/Alder	Sali; HiSG	
*P. deltoides*	Plains cottonwood	Aspen/Alder	Sali; HiSG	
*P. tremuloides*	Quaking aspen	Aspen/Alder	Sali; HiSG	
*P. balsamifera*	Black cottonwood	Aspen/Alder	Sali; LoSG	
*P. fremontii*	Fremont cottonwood	Aspen/Alder	Sali; HiSG	
*P. angustifolia*	Narrlowleaf cottonwood	Aspen/Alder	Sali; HiSG	
*P. alba*	Silver poplar	Aspen/Alder	Sali; HiSG	
*P. nigra*	Lombardy poplar	Aspen/Alder	Sali; HiSG	
*Prosopis spp.*	Mesquite spp.	Woodland	Fab/Jug	Fab/Ros; WL
*P. glandulosa*	Honey mesquite	Woodland	Fab/Jug	Fab/Ros; WL
*P. velutina*	Velvet mesquite	Woodland	Fab/Jug	Fab/Ros; WL
*P. pubescens*	Screwbean mesquite	Woodland	Fab/Jug	Fab/Ros; WL
*Prunus spp.*	Cherry/plum spp.	Mixed HW	Cor/Eri/Lau/Etc	Fab/Ros; WL
*P. pensylvanica*	Pin cherry	Mixed HW	Cor/Eri/Lau/Etc	Fab/Ros; WL
*P. serotina*	Black cherry	Mixed HW	Cor/Eri/Lau/Etc	Fab/Ros; WL
*P. virginiana*	Chokecherry	Mixed HW	Cor/Eri/Lau/Etc	Fab/Ros; WL
*P. persica*	Peach	Mixed HW	Cor/Eri/Lau/Etc	Fab/Ros; WL
*P. nigra*	Canada plum	Mixed HW	Cor/Eri/Lau/Etc	Fab/Ros; WL
*P. americana*	American plum	Mixed HW	Cor/Eri/Lau/Etc	Fab/Ros; WL
*P. emarginata*	Bitter cherry	Woodland	Cor/Eri/Lau/Etc	Fab/Ros; WL
*P. alleghaniensis*	Allegheny plum	Mixed HW	Cor/Eri/Lau/Etc	Fab/Ros; WL
*P. angustifolia*	Chickasaw plum	Mixed HW	Cor/Eri/Lau/Etc	Fab/Ros; WL
*P. avium*	Sweet cherry (domestic)	Mixed HW	Cor/Eri/Lau/Etc	Fab/Ros; WL
*P. cerasus*	Sour cherry (domestic)	Mixed HW	Cor/Eri/Lau/Etc	Fab/Ros; WL
*P. domestica*	European plum (domestic)	Mixed HW	Cor/Eri/Lau/Etc	Fab/Ros; WL
*P. mahaleb*	Mahaleb cherry (domestic)	Mixed HW	Cor/Eri/Lau/Etc	Fab/Ros; WL
*Quercus spp.*	Oak spp.	H Maple/Oak	Faga; Decid	Fagac; WL
*Q. agrifolia*	California live oak	H Maple/Oak	Faga; Evergrn	Fagac; WL
*Q. alba*	White oak	H Maple/Oak	Faga; Decid	Fagac; WL
*Q. arizonica*	Arizona white oak	Woodland	Faga; Decid	Fagac; WL
*Q. bicolor*	Swamp white oak	H Maple/Oak	Faga; Decid	Fagac; WL
*Q. chrysolepis*	Canyon live oak	H Maple/Oak	Faga; Decid	Fagac; WL
*Q. coccinea*	Scarlet oak	H Maple/Oak	Faga; Decid	Fagac; WL
*Q. douglasii*	Blue oak	H Maple/Oak	Faga; Evergrn	Fagac; WL
*Q. sinuata var. sinuata*	Durand oak	H Maple/Oak	Faga; Decid	Fagac; WL
*Q. ellipsoidalis*	Northern pin oak	H Maple/Oak	Faga; Decid	Fagac; WL
*Q. emoryi*	Emory oak	Woodland	Faga; Decid	Fagac; WL
*Q. engelmannii*	Englemann oak	H Maple/Oak	Faga; Decid	Fagac; WL
*Q. falcata*	Southern red oak	H Maple/Oak	Faga; Decid	Fagac; WL
*Q. pagoda*	Cherrybark oak	H Maple/Oak	Faga; Decid	Fagac; WL
*Q. gambelii*	Gambel oak	Woodland	Faga; Decid	Fagac; WL
*Q. garryana*	Oregon white oak	H Maple/Oak	Faga; Decid	Fagac; WL
*Q. ilicifolia*	Scrub oak	H Maple/Oak	Faga; Decid	Fagac; WL
*Q. imbricaria*	Shingle oak	H Maple/Oak	Faga; Decid	Fagac; WL
*Q. kelloggii*	California black oak	H Maple/Oak	Faga; Decid	Fagac; WL
*Q. laevis*	Turkey oak	H Maple/Oak	Faga; Decid	Fagac; WL
*Q. laurifolia*	Laurel oak	H Maple/Oak	Faga; Evergrn	Fagac; WL
*Q. lobata*	California white oak	H Maple/Oak	Faga; Decid	Fagac; WL
*Q. lyrata*	Overcup oak	H Maple/Oak	Faga; Decid	Fagac; WL
*Q. macrocarpa*	Bur oak	H Maple/Oak	Faga; Decid	Fagac; WL
*Q. marilandica*	Blackjack oak	H Maple/Oak	Faga; Decid	Fagac; WL
*Q. michauxi*	Swamp chestnut oak	H Maple/Oak	Faga; Decid	Fagac; WL
*Q. muehlenbergii*	Chinkapin oak	H Maple/Oak	Faga; Decid	Fagac; WL
*Q. nigra*	Water oak	H Maple/Oak	Faga; Decid	Fagac; WL
*Q. texana*	Texas red oak	H Maple/Oak	Faga; Decid	Fagac; WL
*Q. oblongifolia*	Mexican blue oak	Woodland	Faga; Decid	Fagac; WL
*Q. palustris*	Pin oak	H Maple/Oak	Faga; Decid	Fagac; WL
*Q. phellos*	Willow oak	H Maple/Oak	Faga; Decid	Fagac; WL
*Q. prinus*	Chestnut oak	H Maple/Oak	Faga; Decid	Fagac; WL
*Q. rubra*	Northern red oak	H Maple/Oak	Faga; Decid	Fagac; WL
*Q. shumardii*	Shumard oak	H Maple/Oak	Faga; Decid	Fagac; WL
*Q. stellata*	Post oak	H Maple/Oak	Faga; Decid	Fagac; WL
*Q. simili*	Delta post oak	H Maple/Oak	Faga; Decid	Fagac; WL
*Q. velutina*	Black oak	H Maple/Oak	Faga; Decid	Fagac; WL
*Q. virginiana*	Live oak	H Maple/Oak	Faga; Evergrn	Fagac; WL
*Q. wislizeni*	Interier live oak	H Maple/Oak	Faga; Evergrn	Fagac; WL
*Q. margarettiae*	Dwarf post oak	H Maple/Oak	Faga; Evergrn	Fagac; WL
*Q. minima*	Dwarf live oak	H Maple/Oak	Faga; Evergrn	Fagac; WL
*Q. incana*	Bluejack oak	H Maple/Oak	Faga; Decid	Fagac; WL
*Q. hypoleucoides*	Silverleaf oak	Woodland	Faga; Decid	Fagac; WL
*Q. oglethorpensis*	Oglethorpe oak	H Maple/Oak	Faga; Decid	Fagac; WL
*Q. prinoides*	Dwarf chinkapin oak	H Maple/Oak	Faga; Decid	Fagac; WL
*Q. grisea*	Gray oak	Woodland	Faga; Decid	Fagac; WL
*Q. rugosa*	Netleaf oak	H Maple/Oak	Faga; Decid	Fagac; WL
*Q. gracilliformis*	Chisos oak	Woodland	Faga; Decid	Fagac; WL
*Amyris elemifera*	Sea torchwood	Mixed HW	Cor/Eri/Lau/Etc	
*Annona glabra*	Pond apple	Mixed HW	Cor/Eri/Lau/Etc	
*Bursera simaruba*	Gumbo limbo	Mixed HW	Cor/Eri/Lau/Etc	
*Casuarina spp.*	Sheoak spp.	Mixed HW	Cor/Eri/Lau/Etc	
*C. glauca*	Gray sheoak	Mixed HW	Cor/Eri/Lau/Etc	
*C. lepidophloia*	Belah	Mixed HW	Cor/Eri/Lau/Etc	
*Cinnamomum camphora*	Camphortree	Mixed HW	Cor/Eri/Lau/Etc	
*Citharexylum fruticosum*	Florida fiddlewood	Mixed HW	Cor/Eri/Lau/Etc	
*Citrus spp.*	Citrus spp.	Mixed HW	Cor/Eri/Lau/Etc	
*Coccoloba diversifolia*	Tietongue/pigeon plum	Mixed HW	Cor/Eri/Lau/Etc	
*Colubrina elliptica*	Soldierwood	Mixed HW	Cor/Eri/Lau/Etc	
*Cordia sebestena*	Longleaf geigertree	Mixed HW	Cor/Eri/Lau/Etc	
*Cupaniopsis anacardioides*	Carrotwood	Mixed HW	Cor/Eri/Lau/Etc	
*Condalia hookeri*	Bluewood	Woodland	Cor/Eri/Lau/Etc	
*Ebenopsis ebano*	Blackbead ebony	Woodland	Fab/Jug	Fab/Ros; WL
*Leucaena pulverulenta*	Great leadtree	Woodland	Fab/Jug	Fab/Ros; WL
*Sophora affinis*	Texas sophora	Woodland	Fab/Jug	Fab/Ros; WL
*Eugenia rhombea*	Red stopper	Mixed HW	Cor/Eri/Lau/Etc	
*Exothea paniculata*	Butterbough/inkwood	Mixed HW	Cor/Eri/Lau/Etc	
*Ficus aurea*	Florida strangler fig	Mixed HW	Cor/Eri/Lau/Etc	
*Ficus citrifolia*	Banyantree/shortleaf fig	Mixed HW	Cor/Eri/Lau/Etc	
*Guapira discolo*	Beeftree/longleaf blolly	Mixed HW	Cor/Eri/Lau/Etc	
*Hippomane mancinella*	Manchineel	Mixed HW	Cor/Eri/Lau/Etc	
*Lysiloma latisiliquum*	False tamarind	Mixed HW	Fab/Jug	Fab/Ros; WL
*Mangifera indica*	Mango	Mixed HW	Cor/Eri/Lau/Etc	
*Metopium toxiferum*	Florida poisontree	Mixed HW	Cor/Eri/Lau/Etc	
*Piscidia piscipula*	Fishpoison tree	Mixed HW	Fab/Jug	Fab/Ros; WL
*Schefflera actinophylla*	Octopus tree/schefflera	Mixed HW	Cor/Eri/Lau/Etc	
*Sideroxylon foetidissimum*	False mastic	Mixed HW	Cor/Eri/Lau/Etc	
*Sideroxylon salicifolium*	White bully/willow bustic	Mixed HW	Cor/Eri/Lau/Etc	
*Simarouba glauca*	Paradisetree	Mixed HW	Cor/Eri/Lau/Etc	
*Syzygium cumini*	Java plum	Mixed HW	Cor/Eri/Lau/Etc	
*Tamarindus indica*	Tamarind	Mixed HW	Fab/Jug	Fab/Ros; WL
*Robinia pseudoacacia*	Black locust	Mixed HW	Fab/Jug	Fab/Ros; WL
*Robinia neomexicana*	New Mexico locust	Woodland	Fab/Jug	Fab/Ros; WL
*Acoelorraphe wrightii*	Everglades palm	Mixed HW	Cor/Eri/Lau/Etc	
*Coccothrinax argentata*	Florida silver palm	Mixed HW	Cor/Eri/Lau/Etc	
*Cocos nucifera*	Coconut palm	Mixed HW	Cor/Eri/Lau/Etc	
*Roystonea spp.*	Royal palm spp.	Mixed HW	Cor/Eri/Lau/Etc	
*Sabal Mexicana*	Mexican palmetto	Mixed HW	Cor/Eri/Lau/Etc	
*Sabal palmetto*	Cabbage palmetto	Mixed HW	Cor/Eri/Lau/Etc	
*Thrinax morrisii*	Key thatch palm	Mixed HW	Cor/Eri/Lau/Etc	
*Thrinax radiata*	Florida thatch palm	Mixed HW	Cor/Eri/Lau/Etc	
*Arecaceae*	Other palms	Mixed HW	Cor/Eri/Lau/Etc	
*Sapindus saponaria*	Western soapberry	Mixed HW	Cor/Eri/Lau/Etc	
*Salix spp.*	Willow spp.	Aspen/Alder	Sali; HiSG	
*S. amygdaloides*	Peachleaf willow	Aspen/Alder	Sali; HiSG	
*S. nigra*	Black willow	Aspen/Alder	Sali; HiSG	
*S. bebbiana*	Bebb willow	Aspen/Alder	Sali; HiSG	
*S. bonplandiana*	Bonpland willow	Aspen/Alder	Sali; HiSG	
*S. caroliniana*	Coastal plain willow	Aspen/Alder	Sali; HiSG	
*S. pyrifolia*	Balsam willow	Aspen/Alder	Sali; HiSG	
*S. alba*	White willow	Aspen/Alder	Sali; HiSG	
*S. scouleriana*	Scouder’s willow	Aspen/Alder	Sali; HiSG	
*S. sepulcralis*	Weeping willow	Aspen/Alder	Sali; HiSG	
*Sassafras albidum*	Sassafrass	Mixed HW	Cor/Eri/Lau/Etc	
*Sorbus spp.*	Mountain ash spp.	Mixed HW	Cor/Eri/Lau/Etc	Fab/Ros; WL
*S. americana*	American mountain ash	Mixed HW	Cor/Eri/Lau/Etc	Fab/Ros; WL
*S. aucuparia*	European mountain ash	Mixed HW	Cor/Eri/Lau/Etc	Fab/Ros; WL
*S. decora*	Northern mountain ash	Mixed HW	Cor/Eri/Lau/Etc	Fab/Ros; WL
*Swietenia mahagoni*	West Indian mahogany	Mixed HW	Cor/Eri/Lau/Etc	
*Tilia spp.*	Basswood spp.	Mixed HW	Hip/Til	
*T. americana*	American basswood	Mixed HW	Hip/Til	
*T. americana var. heterophylla*	White basswood	Mixed HW	Hip/Til	
*T. americana var. caroliniana*	Carolina basswood	Mixed HW	Hip/Til	
*Ulmus spp.*	Elm spp.	Mixed HW	Cor/Eri/Lau/Etc	
*U. alata*	Winged elm	Mixed HW	Cor/Eri/Lau/Etc	
*U. americana*	American elm	Mixed HW	Cor/Eri/Lau/Etc	
*U. crassifolia*	Cedar elm	Mixed HW	Cor/Eri/Lau/Etc	
*U. pumila*	Siberian elm	Mixed HW	Cor/Eri/Lau/Etc	
*U. rubra*	Slippery elm	Mixed HW	Cor/Eri/Lau/Etc	
*U. serotina*	September elm	Mixed HW	Cor/Eri/Lau/Etc	
*U. thomasii*	Rock elm	Mixed HW	Cor/Eri/Lau/Etc	
*Umbellularia californica*	California laurel	Mixed HW	Cor/Eri/Lau/Etc	
*Yucca brevifolia*	Joshua tree	Mixed HW	Cor/Eri/Lau/Etc	
*Avicennia germinan*	Black mangrove	Mixed HW	Cor/Eri/Lau/Etc	
*Conocarpus erectus*	Button mangrove	Mixed HW	Cor/Eri/Lau/Etc	
*Laguncularia racemosa*	White mangrove	Mixed HW	Cor/Eri/Lau/Etc	
*Rhizophora mangle*	American mangrove	Mixed HW	Cor/Eri/Lau/Etc	
*Olneya tesota*	Desert ironwood	Woodland	Fab/Jug	Fab/Ros; WL
*Tamarix spp.*	Saltcedar	Mixed HW	Cor/Eri/Lau/Etc	
*Melaleuca quinquenervia*	Melaleuca	Mixed HW	Cor/Eri/Lau/Etc	
*Melia azedarach*	Chinaberry	Mixed HW	Cor/Eri/Lau/Etc	
*Triadica sebifera*	Chinese tallowtree	Mixed HW	Cor/Eri/Lau/Etc	
*Vernicia fordii*	Tungoil tree	Mixed HW	Cor/Eri/Lau/Etc	
*Cotinus obovatus*	Smoketree	Mixed HW	Cor/Eri/Lau/Etc	
*Elaeagnus angustifolia*	Russian olive	Mixed HW	Cor/Eri/Lau/Etc	
*Tree broadleaf*	Unknown dead hardwood	Mixed HW	Cor/Eri/Lau/Etc	
*Tree unknown*	Unknown live tree	Mixed HW	Cor/Eri/Lau/Etc	
*C. phaenopyrum*	Washington hawthorn	Mixed HW	Cor/Eri/Lau/Etc	Fab/Ros; WL
*C. succulenta*	Fleshy hawthorn	Mixed HW	Cor/Eri/Lau/Etc	Fab/Ros; WL
*C. uniflora*	Dwarf hawthorn	Mixed HW	Cor/Eri/Lau/Etc	Fab/Ros; WL
*F. berlandieriana*	Berlandier ash	Mixed HW	Olea; LoSG	
*Persea americana*	Avocado	Mixed HW	Cor/Eri/Lau/Etc	
*Ligustrum sinense*	Chinese privet	Mixed HW	Olea; HiSG	
*Q. gravesii*	Graves oak	H Maple/Oak	Faga; Decid	Fagac; WL
*Q. polymorpha*	Mexican white oak	H Maple/Oak	Faga; Decid	Fagac; WL
*Q. buckleyi*	Buckley oak	H Maple/Oak	Faga; Decid	Fagac; WL
*Q. laceyi*	Lacey oak	H Maple/Oak	Faga; Decid	Fagac; WL
*Cordia boissieri*	Anacahuita Texas olive	Mixed HW	Cor/Eri/Lau/Etc	
*Tamarix aphylla*	Athel tamarisk	Mixed HW	Cor/Eri/Lau/Etc	

The first part of the Chojnacky parameter designator is the species group; text after a semicolon indicates the relevant category when more than one set of coefficients is given for a group

*HiSG* the coefficients given for the highest specific gravity in the designated species group, *LoSG* the lowest specific gravity given for a species group, *MedSG* select the coefficients given for the mid-range specific gravity. *WL* select the set of coefficients given for the woodland type. For example, Fagac; WL indicates that the second to the last line of Table 5, Woodland, Fagaceae should be used rather than the coefficients provided for Hardwood; Fagaceae

In order to systematically assign all the biomass estimates presented in Chojnacky et al. [12] to trees in the FIADB (as in this analysis), we present a short set of steps to make this link. Note that these include our interpretation of some of the assignments of species to groups that are not explicit such as some assignments to the woodland groups or allocation to deciduous versus evergreen. These seven steps, which also include application of the revised root component, are the basis for the biomass equation group assignments in Table 2. Note that tables and figures referenced in this list refer to those in Chojnacky et al. [12]:

1. Overall, follow the placement of taxa as suggested within the manuscript (i.e., as in Tables 2, 3, 4, and Figs. 2, 3, and 4).
2. If a tree record is one of the five families (of Table 4) and the tree diameter is measured as diameter at root collar then one of the Table 4 woodland equations applies. Otherwise, if one of the five (Table 4) families and diameter is dbh then use the appropriate equation from Tables 2 or 3. If not one of the five Table 4 families but tree diameter is provided as a root collar measurement, then convert drc to dbh following information provided in Fig. 1 before applying a Table 2 or 3 equation.
3. The calculations for the woodland (Table 4) Cupressaceae (“Cupre; WL”) uses the “2nd juniper” equation from footnote #2 in Table 5.
4. The Fabaceae/Juglandaceae split into the two groups—“Fab/Jug/Carya” and “Fab/Jug”—is according to the genus *Carya* versus all others (i.e., not-*Carya* ).
5. Fagaceae’s deciduous/evergreen split—“Faga; Decid” and “Faga; Evergrn”—sets deciduous as the default. The Fagaceae allocated to evergreen are those five species explicitly listed as evergreen in Table 3 and those identified as evergreen from the USDA PLANTS database [19], which currently includes the addition of three live oak species.
6. The 6-family general equation at the middle of page 136 (in Table 3 of Chojnacky et al. [12])—“Cor/Eri/Lau/Etc”—is assigned trees by family from 3 sources: (a) the six families listed in Table 3; (b) the five additional families noted in the Fig. 3 caption, and (c) any additional formerly unassigned hardwood species.
7. Roots—the Chojnacky estimates use both of the belowground root equations of Table 6 (the sum of the two is generally equivalent to the original Jenkins root component). Note these are dbh-based, so a drc tree should first convert drc-to-dbh according to Fig. 1. Also note, all other (other than root) components of the original Jenkins et al. [1] are applicable here.

### Identifying equivalence between the alternate biomass estimates

Tests of equivalence of the plot level (tonnes carbon per hectare) representation of the Jenkins and Chojnacky groups are included principally as guidance as to where the choice of biomass equations may matter. The analysis does not address relative accuracy of the two alternatives. Specifically, we focused on equivalence tests of the mean difference between the two estimates at the plot, or stand, level according to region and forest type groups. While these are species (group) level equations, any practical effect (of interest) is at plot to landscape to national (carbon reporting) levels. Equivalence tests are appropriate where the questions are more directly “are the groups similar, or effectively the same?” and not so much “are they different?” [20, 21]. This distinction follows from the idea that failure to reject a null hypothesis of no difference between populations does not necessarily indicate that the null hypothesis is true. The essential characteristic of an equivalence test is that the null hypothesis is stated such that the two populations are different [22, 23] which can be viewed as the reverse of the more common approach to hypothesis testing. The specific measure, or threshold, of where two populations can be considered equivalent versus different is set by researchers and a conclusion of not-different, or equivalent, results from rejecting the null hypothesis (that the two are different).

Equivalence tests presented here are paired-sample tests [24, 25] because each sample is based on estimates from each of the Chojnacky and Jenkins groups. Our test statistic is the difference between estimates (Chojnacky minus Jenkins), and we set “equivalence” as a mean difference less than 5 % of the Jenkins-based estimate. Putting our test in terms of the null and alternative hypotheses following the format of publications describing this approach [22, 24], we have:

Null, H_0_: (Chojnacky-Jenkins) <−5 % Jenkins or (Chojnacky-Jenkins) >5 Jenkins

and

Alternative, H_1_: −5 % Jenkins ≤ (Chojnacky-Jenkins) ≤5 % Jenkins

We use the two one-sided tests (TOST) of our two-part null hypothesis that the plot-level difference was greater than 5 % of the Jenkins value and set α = 0.05—one test that the mean difference is less than minus 5 % of the Jenkins estimate, and one test that the mean difference is greater than 5 % of the Jenkins estimate. Within an application of the TOST where α is set to 0.05, a one-step approach to accomplish the TOST result is establish a 2-sided 90 % confidence interval for the test statistic; if this falls entirely within the prescribed interval then the two populations can be considered equivalent [26]. We also extended the level of “equivalence” to within 10 % of the Jenkins-based estimates for some analyses in order to look for more general trends, or broad agreement between the two approaches.

Our equivalence tests are based on the paired estimates of carbon tonnes per hectare on the single-condition forested plots variously classified according to regions described in Fig. 1, forest type-groups listed in Table 1, or stand size class as in Fig. 3 (see [13] for additional details about these classifications). The distribution of the test statistic (mean difference) was obtained from resampling with replacement [27] ten thousand times, with a mean value determined for each sample. The number of plots available varied depending on the classification (Table 1; Fig. 3). We did not test for equivalence if fewer than 30 plots were available, and if over 2000 plots were available we randomly selected 2000 for resampling. The choice of 2000 is based on preliminary analysis of these data that showed the confidence interval from resampling converge with percentiles obtained directly from the distribution of the large number of sample plots, usually well below 1000; the 2000 is simply a round number well beyond this convergence without getting too computationally intense. The 90 % confidence interval (the same as the 95 % interval of TOST) obtained for the distribution of the mean difference is according to a bias corrected and accelerated percentile method [28, 29]. Note that our tests for equivalence are based on comparing this confidence interval to the ±5 % of the corresponding Jenkins based estimate. Table 1 provides the estimates from the two approaches, with the equivalence test results indicated with asterisks. Similarly, the equivalence test results in Fig. 3 are not in the tonnes per hectare of the resampled values and the confidence interval, they are represented as percentage of Jenkins estimates—for this, equivalence is established if the entire confidence interval is within the zero side of the respective 5 %.
